# Eating patterns in Korean adults, 1998–2018: increased energy contribution of ultra-processed foods in main meals and snacks

**DOI:** 10.1007/s00394-023-03258-x

**Published:** 2023-11-24

**Authors:** Sukyoung Jung, Jee Young Kim, Sohyun Park

**Affiliations:** 1 Chungnam National University Hospital Biomedical Research Institute, Daejeon, South Korea; 2 National Food Safety Information Service, Seoul, South Korea; 3 Department of Food Science and Nutrition, Hallym University, Chuncheon, Gangwon 24252 South Korea; 4 The Korean Institute of Nutrition, Hallym University, Chuncheon, South Korea

**Keywords:** Eating patterns, Energy intake, Snacking, Trend, Korea National Health and Nutrition Examination Survey

## Abstract

**Purpose:**

Unfavorable changes in eating patterns over time may contribute to upward trends in chronic diseases, such as obesity. We examined 20-year trends in the percentage of energy from main meals and snacks and the food sources of each eating occasion among Korean adults.

**Methods:**

This study used nationally representative data from the 1st, 4th, and 7th Korea National Health and Nutrition Examination Surveys (1998, 2007–2009, and 2016–2018) among adults aged 20–69 years (*n* = 29,389). Each eating occasion (breakfast, lunch, dinner, and snacks) was defined by respondents during a 24-h dietary recall interview. To identify the food sources of each eating occasion, we used the NOVA system. The percentage of energy at each eating occasion and that from each NOVA group across survey cycles were estimated, and tests for linear trends were conducted using orthogonal polynomial contrasts in linear regression models. All analyses accounted for the complex survey design.

**Results:**

After adjusting for age and sex, the percentage of energy from breakfast decreased from 25.0% in 1998 to 16.7% in 2018 (difference, − 8.2%; standard error [SE], 0.3), whereas that from dinner and snacks increased from 31.1 to 33.8% (difference, + 2.7%; SE, 0.4) and from 14.0 to 19.0% (difference, + 5.0%; SE, 0.5), respectively (all *P* < 0.001). At all eating occasions, the percentage of energy from minimally processed foods declined (difference, − 18.6% for breakfast; − 13.1% for lunch; − 21.1% for dinner; − 20.7% for snacks), while that from ultra-processed foods increased (difference, + 17.0% for breakfast; + 11.3% for lunch; + 18.0% for dinner; + 30.7% for snacks). When stratified by age, the given trends were shown to a greater extent in younger adults (< 50 years old) than in older adults (≥ 50 years old).

**Conclusions:**

The eating patterns of Korean adults changed from 1998 to 2018, with the greatest decrease in energy intake from breakfast and the greatest increase from snacking. At all eating occasions, the contribution of minimally processed foods declined, while that of ultra-processed foods increased, especially among younger adults.

**Supplementary Information:**

The online version contains supplementary material available at 10.1007/s00394-023-03258-x.

## Introduction

Nutrition research has transitioned from individual nutrient- or food-based approaches to meal-based approaches to better understand the effect of the whole diet on health [1]. Although there is no accepted definition, the term “eating patterns” includes frequency of meals, regularity of meals, meal skipping, meal timing, meal food sequencing, nutrient composition, presence of others at a meal, and eating location [1]. Previous studies have shown a link between unfavorable eating patterns (e.g., skipping breakfast, lower meal frequency (≤ 2 meals/day), eating alone, late eating) and poor nutritional quality [2–6] and/or health outcomes, including obesity-related measures [3, 4, 6–13].

Among various eating patterns, characterizing changes in the distribution of eating throughout the day may be helpful to determine whether changes in one eating behavior lead to compensatory changes in other eating behaviors [14, 15]. For example, it was previously reported that increased energy consumption from snacking was compensated for by decreasing energy from main meals in US adults [16]. Additionally, consuming more energy early in the day than later in the day may provide physiological benefits, including decreased inflammation and improved circadian rhythm [17]. Above all, what and how much food is eaten at each eating occasion can be more important. For example, if someone consumes most of their energy from breakfast but mostly from unhealthy foods (e.g., ultra-processed foods), then we cannot conclude that this person follows a healthy eating pattern.

An increase in the frequency of eating occasions, particularly the frequency of snacking, and its subsequent contribution to energy intake have become evident in numerous countries. For instance, data indicate a rise in meal frequency from three to five times a day and an increase in the percentage of energy from snacks between 1971 and 2010 among US adults [14]. In the Chinese population, upward trends in the percentage of energy from snacks were observed between 1991 and 2018 [18]. A prospective cohort study has demonstrated a positive association between self-reported between-meal snacking and a risk of substantial weight gain [19]. Furthermore, in numerous instances, heightened snacking habits have been linked to increased consumption of ultra-processed foods, which, in turn, is associated with an elevated risk of developing obesity and various metabolic disorders [20, 21].

A few studies reported that Koreans have also changed their eating patterns during the last decade. Between 2011 and 2019, consumption of grains, vegetables, fruits, and alcoholic drinks decreased, while that of beverages and meat increased [22]. The proportion of skipping breakfast increased, while that of skipping lunch or dinner largely remained unchanged [22]. Between 2010 and 2018, there were increasing trends in ultra-processed food consumption [23]. However, there is no study characterizing the trends in energy distribution throughout the day including snacking and food sources in Korea.

We examined secular trends in the energy intake contribution of main meals and snacks and the four food groups categorized by NOVA in each main meal and snack among Korean adults aged 20–69 years using the Korea National Health and Nutrition Examination Survey (KNHANES) data from 1998, 2007–2009, and 2016–2018.

## Methods

### Study population

KNHANES is a cross-sectional and nationally representative survey administered by the Korea Disease Control and Prevention Agency to monitor the health and nutritional status of the Korean civilian, noninstitutionalized population using a complex and multistage probability sampling design [24]. KNHANES data are collected through health interviews, health examinations including the collection of biomarkers, and nutrition surveys. More details about KNHANES data collection methods have been published elsewhere [24]. The present study combines data from the 1st (1998), 4th (2007–2009), and 7th (2016–2018) KHANES.

Among 32,228 adults aged 20–69 years who completed both health interviews and nutrition surveys, we excluded those with the following conditions: implausible energy intake based on < 1st or > 99th percentile (< 500 or > 5000 kcal/day) (*n* = 1125); pregnancy or breastfeeding (*n* = 255); and severe chronic diseases, including heart diseases, cancer, liver cirrhosis, and renal failure (*n* = 1459). The final analytic sample included 29,389 adults (12,329 males and 17,060 females) (Supplemental Fig. 1).

### Data collection

In mobile examination centers, trained interviewers conducted health interviews to collect detailed information on sociodemographic characteristics, health-related behaviors, and morbidity.

In participants’ homes, trained dieticians administered a 24-h dietary recall interview 1 week after the health interviews and examinations. Participants reported on all foods and beverages consumed in the previous 24 h, including the description, quantity (in units of volume), and time and place of eating. To minimize respondent burden and to obtain an accurate food recall, the multiple-pass approach was used with the assistance of tools such as two-dimensional measuring guides and/or standard measuring tools. For the home-cooked dishes, the person in charge of cooking reported the details of his or her home recipes.

#### Energy and nutrient intake assessment

Daily total energy and nutrient intakes were estimated by multiplying the intakes of all foods and beverages reported by participants and publicly available in the KNAHNES dietary data. When calculating total energy and nutrient intake, the Korean Food Composition Table of the Rural Development Administration was used (5th edition for 1998; 7th edition for 2007–2009, 9th edition for 2016–2018) [25]. Our main interest was total energy intake (kcal).

### Assessment of outcome

#### Eating occasion

Each eating occasion was defined based on eating events named by respondents during the 24-h dietary recall interview. There were four eating occasion response options for respondents to choose from: breakfast, lunch, dinner, and snacks. The percentage of energy (% kcal) at each eating occasion was used as a main outcome. If the given eating occasion was skipped, the % kcal at that eating occasion was set to 0. For example, the % kcal of breakfast would be 0 for an individual who skipped breakfast.

#### Food sources for each eating occasion using the Korean NOVA system

To identify the major food sources of each main meal and snack, we used a 5-digit food code to categorize all food items by NOVA. NOVA enabled us to assess diets according to the extent and purpose of food processing [26]. In NOVA, there are four food groups, including unprocessed or minimally processed foods (Group 1), processed culinary ingredients (Group 2), processed foods (Group 3), and ultra-processed foods (Group 4) [26].

In general, the principle suggested by Monteiro and colleagues [26] was applied to the overall food classification in this study. We first identified the main ingredients of the food, and if processed, we categorized them into either Group 3 or Group 4, with a focus on the maintenance of the food matrix. However, the NOVA system developed in Brazil may not be directly applicable to Korean cuisine, which is characterized by dishes that combine many ingredients. Thus, we employed a Korean NOVA system that adheres to the rationale of the NOVA classification but specifically considers the preservation of the natural food matrix and traditional eating experiences for application to Korean cuisine. If a dish was not clearly distinguishable between Group 3 and Group 4 based on its ingredients, another criterion was used whether the dish was part of the traditional Korean diet before the mass production of processed foods [27]. Additionally, we used food codes in the KNHANES dataset to identify traditional ingredients, such as Korean fermented sauces like soybean paste. According to the original NOVA system [26], soybean paste can be classified as an ultra-processed food. In contrast, in the Korean NOVA system, it could be classified as either processed or ultra-processed depending on whether it is produced traditionally or through mass production. This distinction was made by checking the food codes in the KNHANES dataset, which uses separate codes for homemade and factory-made products. Detailed examples are described elsewhere [27].

Two investigators (S.J. and JY. K.) classified all consumed food and beverage items at the food or ingredient level into one of the four NOVA food groups. In cases of disagreements in classification, we (1) identified the raw materials by checking product names and manufacturer information, and (2) reviewed the nutrient database to ascertain the sodium or sugar contents of such items [28]. During the 24-h dietary recall interview conducted in each survey cycle, participants reported consuming 1453 items in 1998, 2725 items in 2007–2009; and 3894 items in 2016–2018, respectively. Unprocessed or minimally processed foods included raw vegetables, fruits, meats, grains (557 items in 1998; 798 items in 2007–2009; 966 in 2016–2018). Processed culinary ingredients comprised condiments and oils (34 items in 1998; 147 items in 2007–2009; 247 items in 2016–2018). Processed foods include soups, stew, kimchi, and grilled or marinated vegetables and meat (275 items in 1998; 334 items in 2007–2009; 504 items in 2016–2018). Ultra-processed foods included bread, snacks, distilled alcoholic beverages, soft drinks, and noodles (587 items in 1998; 1446 items in 2007–2009; 2177 items in 2016–2018). Supplemental Table 1 shows the most frequently consumed food or ingredient items for each NOVA group across the survey cycle for each eating occasion. For each eating occasion, the percentage of energy (% kcal) from each NOVA group was calculated and used for further analysis.

### Assessment of covariates

Covariates included age (years), sex (males, females), residency (urban, rural), education level (elementary school, middle school, high school, college and higher), household income (quartiles), and marital status (married or not) for presenting the general characteristics of study participants. For stratification analysis, trends in the percentage of energy and the food sources from all eating occasions were further assessed among population subgroups by age: 20–29, 30–39, 40–49, 50–59, and 60–69 years old.

### Statistical analysis

Sociodemographic characteristics of Korean adults by survey cycle were presented as weighted proportions with standard errors (SEs). After adjusting for age and sex, linear regression models were used to estimate (1) the percentage of energy at each eating occasion and (2) the percentage of energy from each NOVA group for each eating occasion across the survey cycle. Statistical significances of linear trends over time were examined with the survey cycle modeled as an orthogonal polynomial [29]. A potential interaction effect by age was checked using an interaction term between survey cycle and age in linear regression models, and age-stratified estimates were obtained.

Survey weights accounting for the complex sampling design of the KNHANES were applied to all analyses. All statistical analyses were conducted with SAS software (version 9.4, SAS Institute, Cary, NC, USA) using PROC SURVEY procedures, with significance determined at an *α* level of 0.05.

## Results

### Population characteristics

From 1998 to 2018, the mean age of respondents increased from 39.4 to 43.4 years old. The proportion of adults aged 20–39 years decreased from 56.2 to 40.4%, whereas those aged 40–69 years increased from 43.9 to 59.6%. The proportion of urban residency increased from 77.9 to 87.9%. The proportion of respondents with an education level of college graduate or higher increased from 27.5 to 48.0%. The proportion of married respondents decreased from 73.9 to 66.8% (Table 1).

**Table 1:** Demographic characteristics of Korean adults aged 20–69 years by KNHANES cycle (*n* = 29,389)^a^

	Overall (*n* = 29,389)	1st KNHANES, 1998 (*n* = 5748)	4th KNHANES, 2007–2009 (*n* = 11,900)	7th KNHANES, 2016–2018 (*n* = 11,741)
Age, years	42.2 (0.1)	39.4 (0.3)	41.6 (0.2)	43.4 (0.2)
Age group, years				
20–29	21.2 (0.4)	27.2 (0.9)	21.3 (0.7)	19.6 (0.6)
30–39	23.5 (0.4)	29.0 (1.1)	25.0 (0.7)	20.8 (0.6)
40–49	24.1 (0.4)	20.8 (0.8)	25.2 (0.6)	24.1 (0.6)
50–59	19.6 (0.3)	13.4 (0.6)	17.8 (0.5)	22.6 (0.5)
60–69	11.6 (0.2)	9.7 (0.6)	10.6 (0.3)	12.9 (0.4)
Sex				
Males	51.0 (0.3)	50.4 (0.5)	51.1 (0.5)	51.1 (0.5)
Females	49.0 (0.3)	49.6 (0.5)	48.9 (0.5)	48.9 (0.5)
Residency				
Urban	84.7 (0.9)	77.9 (1.3)	82.9 (1.6)	87.9 (1.3)
Rural	15.3 (0.9)	22.1 (1.3)	17.1 (1.6)	12.1 (1.3)
Education level				
Elementary school	11.6 (0.3)	18.1 (0.9)	14.2 (0.4)	7.6 (0.3)
Middle school	9.5 (0.2)	13.0 (0.6)	10.6 (0.4)	7.6 (0.3)
High school	39.9 (0.5)	41.3 (1.0)	43.2 (0.7)	36.7 (0.7)
College and higher	39.1 (0.6)	27.5 (1.3)	32.0 (0.8)	48.0 (0.9)
Household income				
Q1	11.6 (0.3)	16.8 (1.0)	11.9 (0.5)	10.0 (0.5)
Q2	23.9 (0.5)	21.6 (1.1)	25.3 (0.7)	23.4 (0.7)
Q3	31.1 (0.5)	32.1 (1.2)	30.7 (0.7)	31.1 (0.7)
Q4	33.5 (0.6)	29.5 (1.5)	32.1 (1.0)	35.5 (0.9)
Married^b^	69.5 (0.5)	73.9 (1.0)	71.6 (0.8)	66.8 (0.8)

*KNHANES* Korea National Health and Nutrition Examination Survey

^a^All results are weighted and presented as the mean and standard error (SE) for continuous variables and percentage and SE for categorical variables

^b^Married included participants who were married and cohabiting

### Trends in energy intake contribution of main meals and snacks

From 1998 to 2018, there was a significant upward trend in total energy intake; however, the estimates were not substantially different (1986 to 2048 kcal; difference, + 62 kcal [SE, 21]; *P* = 0.004). During the same period, the percentage of energy from breakfast decreased from 25.0 to 16.7% (difference, − 8.2% [SE, 0.3]; *P* = 0.004); that from lunch did not substantially change (29.9–30.5%; difference, + 0.6% [SE, 0.4]; *P* = 0.12); that from dinner increased from 31.1 to 33.8% (difference, + 2.7% [SE, 0.4]; *P* < 0.0001); and that from snacks increased from 14.0 to 19.0% (difference, + 5.0% [SE, 0.5]; *P* < 0.0001) (Table 2).

**Table 2:** Trends in the percentage of energy intake from main meals and snacks among Korean adults aged 20 to 69 years by KNHANES cycle

	Weighted mean (SE)^a^			Mean difference, 7th vs. 1st	% difference, 7th vs. 1st	*P* trend^b^ linear
	1998 (1st)	2007–2009 (4th)	2016–2018 (7th)			
Total energy intake (kcal)	1986 (19)	1932 (10)	2048 (10)	62 (21)	3.1	0.004
% Energy intake from breakfast	25.0 (0.3)	20.8 (0.2)	16.7 (0.2)	− 8.2 (0.3)	− 32.8	< 0.0001
% Energy intake from lunch	29.9 (0.3)	30.7 (0.2)	30.5 (0.2)	0.6 (0.4)	2.0	0.12
% Energy intake from dinner	31.1 (0.3)	31.4 (0.2)	33.8 (0.2)	2.7 (0.4)	8.7	< 0.0001
% Energy intake from snack	14.0 (0.4)	17.2 (0.2)	19.0 (0.2)	5.0 (0.5)	35.7	< 0.0001

*KNHANES* National Health and Nutrition Examination Survey, *SE* standard error

^a^Estimated mean and SE were obtained using the linear regression model after adjusting for age and sex

^b^*P* values for trends were estimated with the survey cycles modeled as an orthogonal polynomial

### Trends in energy intake contribution of NOVA groups for main meals and snacks

**Fig. 1:**
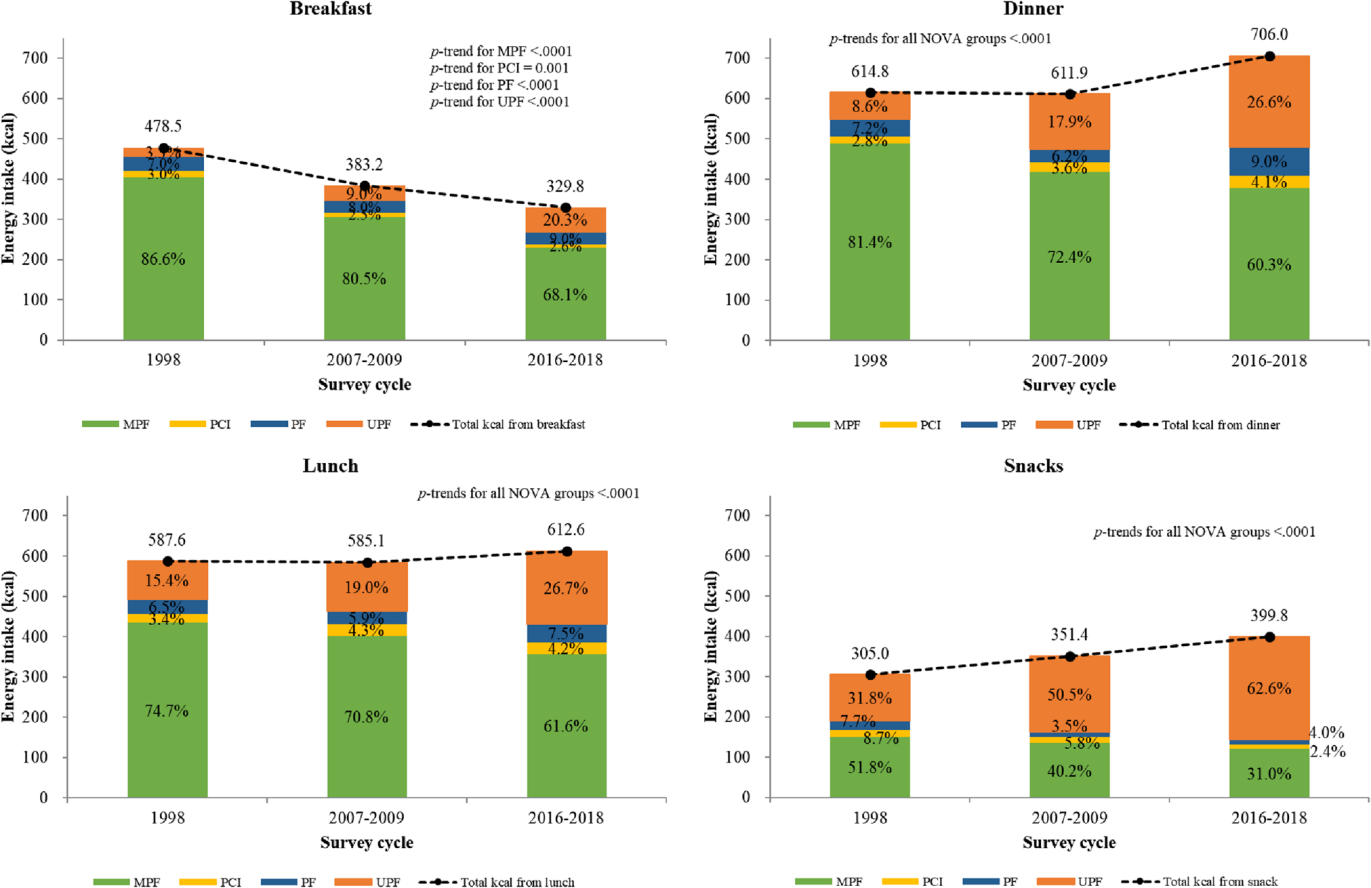
Trends in the percentage of energy intake from NOVA groups for main meals and snacks by KNHANES cycle, Korean adults aged 20–69 years. *MPF* unprocessed or minimally processed foods, *PCI* processed culinary ingredients, *PF* processed foods, *UPF* ultra-processed foods, *KNHANES* National Health and Nutrition Examination Survey, *SE* standard error. Note: The estimates were obtained after adjusting for age and sex using the linear regression model. *P* values for trends were estimated with the survey cycles modeled as an orthogonal polynomial

From 1998 to 2018, at breakfast, the percentage of energy from minimally processed foods decreased from 86.6 to 68.1% (difference, − 18.6% [SE, 0.6]; *P* < 0.0001), whereas that from ultra-processed foods increased from 3.3 to 20.3% (difference, + 17.0% [SE, 0.6]; *P* < 0.0001). At lunch, the percentage of energy from minimally processed foods decreased from 74.7 to 61.6% (difference, − 13.1% [SE, 0.7]; *P* < 0.0001), whereas that from ultra-processed foods increased from 15.4 to 26.7% (difference, + 11.3% [SE, 0.7]; *P* < 0.0001). At dinner, the percentage of energy from minimally processed foods decreased from 81.4 to 60.3% (difference, − 21.1% [SE, 0.6]; *P* < 0.0001), whereas that from ultra-processed foods increased from 8.6 to 26.6% (difference, + 18.0% [SE, 0.6]; *P* < 0.0001). The percentage of energy from snacking on minimally processed foods decreased from 51.8 to 31.0% (difference, − 20.7% [SE, 1.0]; *P* < 0.0001), whereas that from ultra-processed foods increased from 31.8 to 62.6% (difference, + 37.0% [SE, 0.9]; *P* < 0.0001). At all eating occasions, processed culinary ingredients and processed foods contributed the least to total energy intake, and the percentage of energy from those foods did not significantly change (Fig. 1).

### Trends in different age groups

**Fig. 2:**
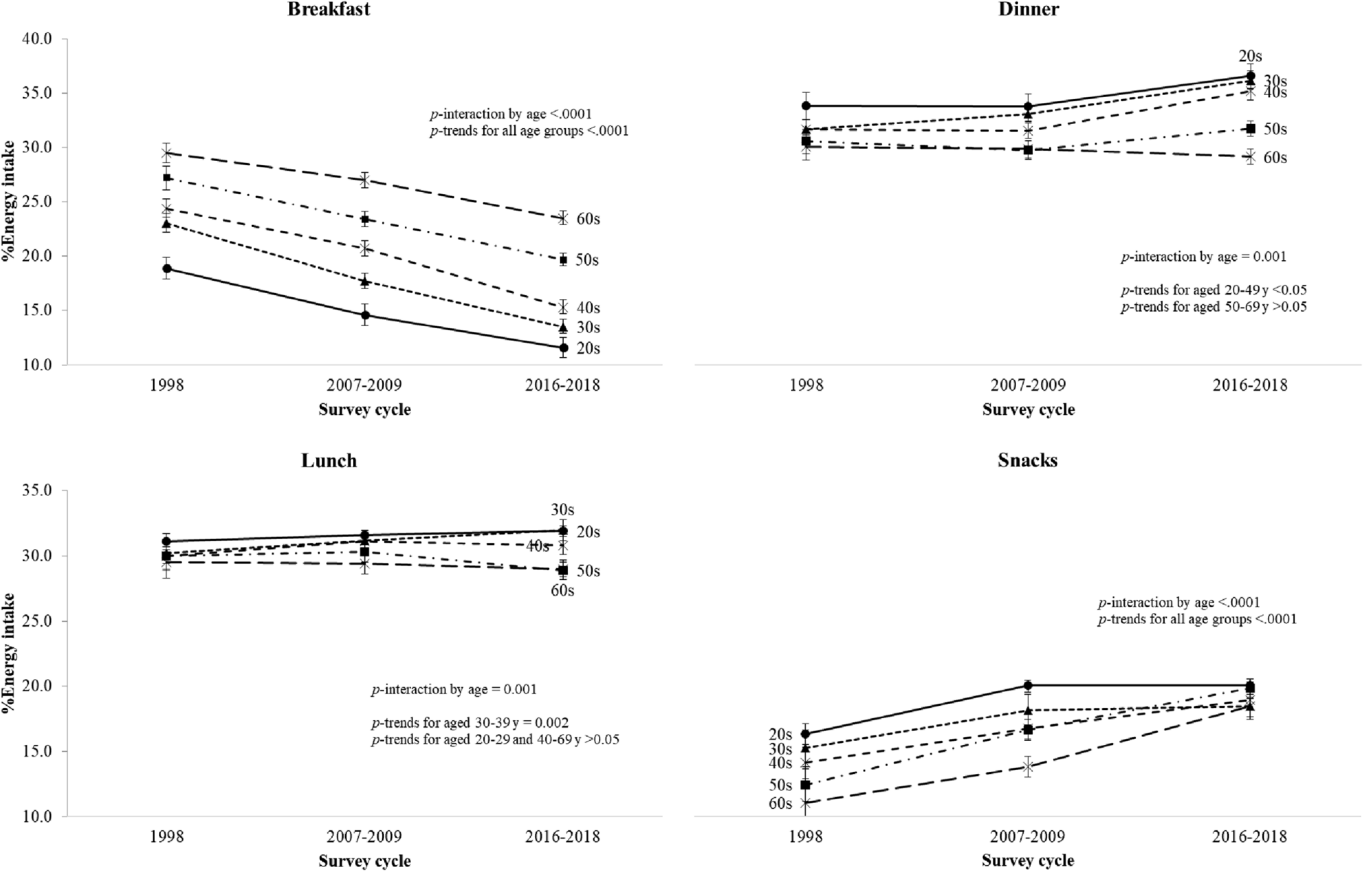
Trends in the percentage of energy intake from main meals and snacks by KNHANES cycle, Korean adults aged 20–69 years, stratified by age. *KNHANES* National Health and Nutrition Examination Survey, *SE* standard error. Note: The estimated mean and SE were obtained after adjusting for sex using the linear regression model. *P* values for trends were estimated with the survey cycles modeled as an orthogonal polynomial.* P* values for interaction were determined by including the interaction term of survey cycle and age in the linear regression model

When compared to their counterparts, adults aged 20–49 years showed greater changes in the percentage of energy from breakfast (difference, − 7.3% for those aged 20–29 years; − 9.5% for those aged 30–39 years; − 9.1% for those aged 40–49 years; − 7.5% for those aged 50–59 years; − 6.0% for those aged 60–69 years; all *P* < 0.0001) and that from dinner (difference, + 2.8% for those aged 20–29 years; + 4.5% for those aged 30–39 years; + 3.5% for those aged 40–49 years; + 1.3% for those aged 50–59 years; − 0.9% for those aged 60–69 years; all *P* < 0.05 except for those aged 50–69 years), while adults aged 50–69 years showed a greater upward trend in the percentage of energy intake from snacks (difference, + 3.7% for those aged 20–29 years; + 3.2% for those aged 30–39 years; + 4.8% for those aged 40–49 years; + 7.4% for those aged 50–59 years; + 7.4% for those aged 60–69 years; all *P* < 0.0001 (Fig. 2).

**Fig. 3:**
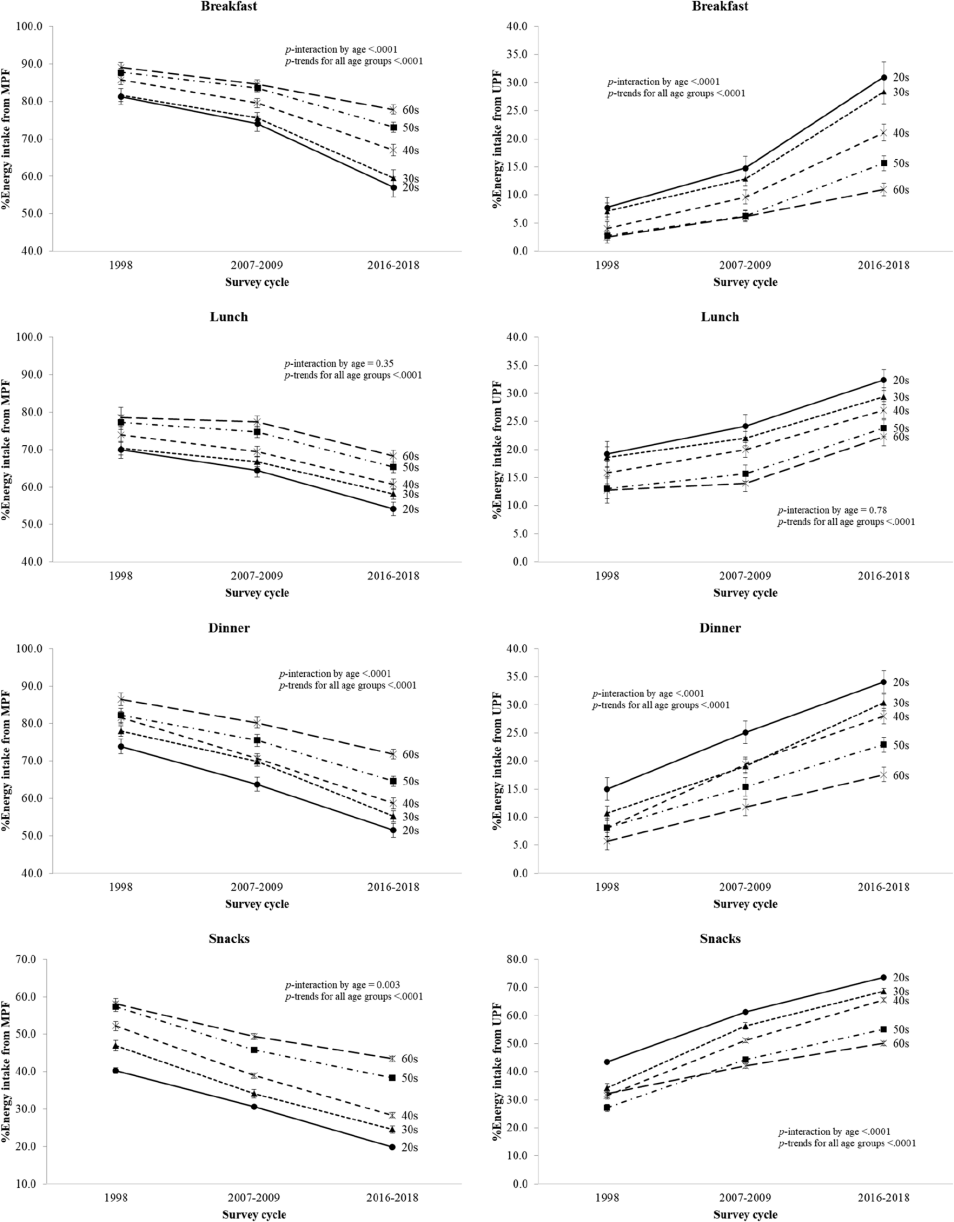
Trends in the percentage of energy intake from minimally processed and ultra-processed foods for main meals and snacks by KNHANES cycle, Korean adults aged 20–69 years, stratified by age. *MPF* unprocessed or minimally processed foods, *UPF* ultra-processed foods, *KNHANES* National Health and Nutrition Examination Survey, *SE* standard error. Note: The estimates were obtained after adjusting for sex using the linear regression model. *P* values for trends were estimated with the survey cycles modeled as an orthogonal polynomial.* P* values for interaction were determined by including the interaction term of survey cycle and age in the linear regression model

At all eating occasions except for lunch, adults aged 20–49 years had a greater decline in the percentage of energy from minimally processed foods (*breakfast*: difference, − 24.2% for those aged 20–29 years; − 22.1% for those aged 30–39 years; − 18.9% for those aged 40–49 years; − 14.7% for those aged 50–59 years; − 11.3% for those aged 60–69 years; all *P* < 0.0001; *dinner*: difference, − 22.4% for those aged 20–29 years; − 22.8% for those aged 30–39 years; − 23.0% for those aged 40–49 years; − 17.6% for those aged 50–59 years; − 14.7% for those aged 60–69 years; all *P* < 0.0001; *snacks*: difference, − 20.4% for those aged 20–29 years; − 22.4% for those aged 30–39 years; − 23.8% for those aged 40–49 years; − 18.9% for those aged 50–59 years; − 14.7% for those aged 60–69 years; all *P* < 0.0001) and a greater increase in the percentage of energy from ultra-processed foods than those aged 50–69 years (*breakfast*: difference, + 23.2% for those aged 20–29 years; + 21.3% for those aged 30–39 years; + 17.1% for those aged 40–49 years; + 12.9% for those aged 50–59 years; + 8.5% for those aged 60–69 years; all *P* < 0.0001; *dinner*: difference, + 19.2% for those aged 20–29 years; + 19.7% for those aged 30–39 years; + 19.9% for those aged 40–49 years; + 14.8% for those aged 50–59 years; + 11.8% for those aged 60–69 years; all *P* < 0.0001; *snacks*: difference, + 30.1% for those aged 20–29 years; + 34.5% for those aged 30–39 years; + 34.2% for those aged 40–49 years; + 27.9% for those aged 50–59 years; + 18.0% for those aged 60–69 years; all *P* < 0.0001) (Fig. 3).

## Discussion

From 1998 to 2018, the percentage of energy from breakfast decreased, but that from dinner and snacks increased. This may indicate that a decreased percentage of energy from breakfast was compensated for by an increased percentage of energy from snacks. At all eating occasions, we observed a decline in the percentage of energy from minimally processed foods and an increase in that from ultra-processed foods, indicating that ultra-processed foods have become major sources of total energy intake. When stratified by age, younger adults (< 50 years old) had a greater decline in the percentage of energy from breakfast and an increase in that from dinner, whereas older adults (≥ 50 years old) had a greater increase in the percentage of energy from snacks than their counterparts; younger adults (< 50 years old) had a steeper decrease in the percentage of energy from minimally processed foods and an increase in that from ultra-processed foods for breakfast, dinner, and snacks compared to older adults (≥ 50 years old).

This study using KNHANES data is the first attempt to examine temporal trends in the energy intake contribution of all eating occasions among Korean adults. Globally, eating patterns have shifted toward a more westernized style, which could be harmful to human health [30]. In our study, the percentage of energy from breakfast significantly decreased, while that from lunch almost remained unchanged and that from dinner slightly increased from 1998 to 2018. This finding is in accordance with a previous study that reported increases in the percentage of skipping breakfast but not noticeable changes in the percentages of skipping lunch and dinner between 2011 and 2019 (skipping breakfast: 21.4% in 2011, 31.3% in 2019; skipping lunch: 7.6% in 2011, 8.0% in 2019; and skipping dinner: 4.8% in 2011, 5.5% in 2019) [22].

Our results showed that a reduced proportion of breakfast appeared to be compensated for by increasing snack consumption. From 1998 to 2018, the percentage of energy from breakfast decreased by 32.8% and that from snacks increased by 35.8%, while total energy intake did not change substantially. Furthermore, breakfast contributed more energy than snacks did in 1998 (25.0% from breakfast vs. 14.0% from snacks); however, it reversed in 2016–2018 (16.7% from breakfast vs. 19.0% from snacks). Despite the varied definition of snacking by study (e.g., snacking frequency or energy contribution from snacking), there is strong evidence that snacking contributes to positive energy balance [31, 32]. Yet, it is not confirmed if snacking per se adversely contributes to health outcomes [31, 32] or nutritional quality [33], but even snacking can be helpful in the older population [33]. More importantly, healthy snacks like whole grains, fruits, and vegetables are beneficial to health; however, popular snacks are usually energy dense and nutrient poor (e.g., salty snacks, sweetened beverages) [31]. Thus, the types of foods for snacks need to be given more attention.

Globally and in Korea, more highly processed foods have become popular [23, 34]. Although food processing per se is not necessarily harmful, ultra-processed foods are generally characterized by poor nutritional profiles and corresponding detrimental health outcomes [34]. From 2011 to 2018, the consumption of fruits and vegetables (i.e., minimally processed) decreased, while that of beverages (i.e., ultra-processed) increased in Korea [22]. Furthermore, coke was newly ranked in the ten most frequently consumed foods in Korea in 2019 [35]. Our study showed that the percentage of energy from ultra-processed foods substantially increased, while that from minimally processed foods decreased from 1998 to 2018 at all eating occasions. Such trends imply that eating patterns regarding food sources among Koreans have worsened during the past 20 years. Thus, applicable recommendations are necessary to improve their eating patterns by highlighting the types of foods that should be consumed (e.g., limiting the consumption of ultra-processed foods).

Age differences were evident in our study, indicating that relatively younger adults had greater changes than older adults. They tended to reduce their energy consumption from minimally processed foods but increase it from ultra-processed foods. These trends may account in part for the greater increase in obesity prevalence among younger adults than older adults between 2009 and 2019 in Korea [36]. Between 2010–2012 and 2016–2018, upward trends in the energy consumption of ultra-processed foods were stronger in younger adults than in older adults (+ 2.5% for those aged 20–49 years; + 1.6% for those aged 50–64 years; + 1.0% for those aged 65 years) [23]. Generally, the majority of people have experienced several life events in their adulthood, including undertaking higher education, getting a job, or becoming a parent. The given life events may require independent living, and obtaining food and preparing meals are their own responsibility in this case [37]. According to a previous study, younger adults are more likely to purchase less expensive, energy-dense, and ready-to-eat foods and less likely to plan their meals, especially under stressful situations (e.g., after a long day at work, examination) [37]. Additionally, they might think that healthy foods are expensive, and they tend to adopt energy-dense diets faster than older adults [38].

Currently, the prevailing recommendations in Korea do not include specific guidelines regarding the consumption of processed foods [39]. Given the unfavorable effect of high consumption of ultra-processed foods on health, it is crucial to continuously monitor consumption patterns and conduct further investigations to establish a causal relationship between the intake of ultra-processed foods and health outcomes. These efforts are necessary to generate compelling evidence that can inform more effective dietary recommendations. Also, our study showed that the increase in ultra-processed food is more evident among younger adults than older adults. To address this issue, several public health strategies could be pursued, particularly targeting the younger adult population. These strategies may include the development of education programs, the implementation of informative package labelling, the modification of the food environment, and the consideration of taxation measures [40].

This study has strengths. To our knowledge, this is the first study to characterize temporal trends in the energy intake contribution and food sources of meals and snacks in Korean adults. Another strength of this study is the use of nationally representative dietary data collected using standardized protocols and providing generalizability to Korean adults.

Several limitations should be noted. First, there are inevitable measurement errors (e.g., underreporting of energy intake) in self-reported dietary assessments, including the 24-h dietary recall interview. Especially in Korean adults, there is a 12% underreporting of total energy intake estimated by 24-h dietary recall when compared with the double-labeled water method [41], and underreporting is likely more prevalent among women living alone and with a lower education level than their counterparts [42]. Furthermore, snacks (especially afternoon snacks) are most likely to be underreported [43]; thus, cautious interpretations are needed. Further studies are required to examine whether underreporting has changed over time and to adjust errors due to underreporting in Korea. Second, the use of only a single 24-h dietary recall may not be sufficient to measure individual usual intakes because of day-to-day variations (a source of within-person random error) [46]. Usual dietary intake is often recommended in some cases (e.g., when examining the proportion of the population at or below a certain level of intake). However, a single 24-h dietary recall is sufficient to estimate the mean intake of a population [44]. Third, the use of respondent descriptions for eating occasions may not be interchangeable with those from other studies since there is no consensus definition of eating occasions [45]. Furthermore, there is a lack of objective methods, such as the examination of biomarkers of eating patterns, to determine whether eating patterns in our study were valid. Fourth, we focused on Korean adults aged 20–69 years, and thus, our findings may not be generalizable to other populations with different races/ethnicities, ages (e.g., adolescents), or locations. Fifth, the NOVA classification system has a few unresolved issues, including that its simplistic definition may ignore the features of food itself [46]. Several types of ultra-processed foods, also known as premium ultra-processed foods (e.g., granola with probiotics, brown bread, or plain yogurt), are not necessarily harmful to health. However, it has been considered the most prominent method of food classification considering the extent and purpose of industrial food processing, which may have been overlooked in previous literature focusing only on nutrient profiles [47]. An updated NOVA classification system may be needed to address this issue. Lastly, to discern the consumption of UPFs, the 5-digit food codes were used from the KNHANES data. It is important to note that using this method may not ensure a precise distinction between Group 3 and Group 4 in the NOVA classification due to the reliance on general food codes. In the KNHANES dataset, processed foods lacking specific brand and product names, as well as food-away-from-home items without listed ingredients, are coded using these general food codes. This approach might lead to an underestimation of individuals’ UPF consumption.

In conclusion, the eating patterns of Korean adults changed from 1998 to 2018, with the greatest decrease in the percentage of energy from breakfast and the greatest increase in the percentage of energy from snacks. On all eating occasions, the contribution of minimally processed foods decreased, while that of ultra-processed foods increased, especially among younger adults. These findings highlight the importance of continuous monitoring of both eating patterns and the types of foods selected for all eating occasions to provide more realistic dietary recommendations that reflect the current state of diets and to offer an opportunity for health improvement. Furthermore, additional studies into the factors contributing to such trends are necessary to better understand the context in which negative changes occurred.

### Supplementary Information

Below is the link to the electronic supplementary material.

Supplementary file1 (DOCX 57 KB)

## Data Availability

The KNHANES data are publically available for download.

## References

[1] Leech RM, Worsley A, Timperio A, McNaughton SA (2015). Understanding meal patterns: definitions, methodology and impact on nutrient intake and diet quality. Nutr Res Rev.

[2] Min C, Noh H, Kang YS, Sim HJ, Baik HW, Song WO, Yoon J, Park YH, Joung H (2011). Skipping breakfast is associated with diet quality and metabolic syndrome risk factors of adults. Nutr Res Pract.

[3] Chung SJ, Lee Y, Lee S, Choi K (2015). Breakfast skipping and breakfast type are associated with daily nutrient intakes and metabolic syndrome in Korean adults. Nutr Res Pract.

[4] Kim S, Park GH, Yang JH, Chun SH, Yoon HJ, Park MS (2014). Eating frequency is inversely associated with blood pressure and hypertension in Korean adults: analysis of the Third Korean National Health and Nutrition Examination Survey. Eur J Clin Nutr.

[5] Chae W, Ju YJ, Shin J, Jang SI, Park EC (2018). Association between eating behaviour and diet quality: eating alone vs. eating with others. Nutr J.

[6] Shim JS, Kim HC (2021). Late eating, blood pressure control, and cardiometabolic risk factors among adults with hypertension: results from the Korea National Health and Nutrition Examination Survey 2010–2018. Epidemiol Health.

[7] Jung CH, Lee JS, Ahn HJ, Choi JS, Noh MY, Lee JJ, Lee EY, Lim JH, Lee YR, Yoon SY, Kim CH, Cho DH, Choi YS, Choi KM (2017). Association of meal frequency with metabolic syndrome in Korean adults: from the Korea National Health and Nutrition Examination Survey (KNHANES). Diabetol Metab Syndr.

[8] Kim YJ, Yoon JH, Choi HS, Kim CS, Bae EH, Ma SK, Kim SW (2020). Meal frequency and skipping breakfast are associated with chronic kidney disease. Nutrients.

[9] Ha K, Song Y (2019). Associations of meal timing and frequency with obesity and metabolic syndrome among Korean adults. Nutrients.

[10] Jeong D, Kim J, Lee H, Kim D-Y, Lim H (2020). Association of cardiometabolic multimorbidity pattern with dietary factors among adults in South Korea. Nutrients.

[11] Jung J, Kim A-S, Ko H-J, Choi H-I, Hong H-E (2020). Association between breakfast skipping and the metabolic syndrome: the Korea National Health and nutrition examination survey, 2017. Medicina.

[12] Kwon AR, Yoon YS, Min KP, Lee YK, Jeon JH (2018). Eating alone and metabolic syndrome: a population-based Korean National Health and Nutrition Examination Survey 2013–2014. Obes Res Clin Pract.

[13] Son H, Kim H (2019). Influence of living arrangements and eating behavior on the risk of metabolic syndrome: a national cross-sectional study in South Korea. Int J Environ Res Public Health.

[14] Kant AK, Graubard BI (2015). 40-year trends in meal and snack eating behaviors of American adults. J Acad Nutr Diet.

[15] Dhurandhar EJ (2016). True, true, unrelated? A review of recent evidence for a causal influence of breakfast on obesity. Curr Opin Endocrinol Diabetes Obes.

[16] Kant AK, Graubard BI (2019). Within-person compensation for snack energy by US adults, NHANES 2007–2014. Am J Clin Nutr.

[17] Paoli A, Tinsley G, Bianco A, Moro T (2019). The influence of meal frequency and timing on health in humans: the role of fasting. Nutrients.

[18] Song X, Wang H, Su C, Wang Z, Zhang J, Ding G, Zhang B (2022). Secular trends in time-of-day of energy intake in a Chinese cohort. Nutrients.

[19] Bes-Rastrollo M, Sanchez-Villegas A, Basterra-Gortari FJ, Nunez-Cordoba JM, Toledo E, Serrano-Martinez M (2010). Prospective study of self-reported usual snacking and weight gain in a Mediterranean cohort: the SUN project. Clin Nutr.

[20] Mendonça RD, Pimenta AM, Gea A, de la Fuente-Arrillaga C, Martinez-Gonzalez MA, Lopes AC, Bes-Rastrollo M (2016). Ultraprocessed food consumption and risk of overweight and obesity: the University of Navarra Follow-Up (SUN) cohort study. Am J Clin Nutr.

[21] Srour B, Fezeu LK, Kesse-Guyot E, Allès B, Méjean C, Andrianasolo RM, Chazelas E, Deschasaux M, Hercberg S, Galan P, Monteiro CA, Julia C, Touvier M (2019). Ultra-processed food intake and risk of cardiovascular disease: prospective cohort study (NutriNet-Santé). BMJ.

[22] Yun S, Oh K (2022). Dietary habits among Korean population. Public Health Wkly Rep PHWR.

[23] Shim JS, Shim SY, Cha HJ, Kim J, Kim HC (2021). Socioeconomic characteristics and trends in the consumption of ultra-processed foods in Korea from 2010 to 2018. Nutrients.

[24] Kweon S, Kim Y, Jang MJ, Kim Y, Kim K, Choi S, Chun C, Khang YH, Oh K (2014). Data resource profile: the Korea National Health and Nutrition Examination Survey (KNHANES). Int J Epidemiol.

[25] Park J, Yeo Y, Yoo JH (2022). Dietary intake and nutritional status in young and middle-aged adults according to the meal frequency from the Korea National Health and Nutritional Survey. Korean J Fam Med.

[26] Monteiro CA, Cannon G, Levy RB, Moubarac JC, Louzada ML, Rauber F, Khandpur N, Cediel G, Neri D, Martinez-Steele E, Baraldi LG, Jaime PC (2019). Ultra-processed foods: what they are and how to identify them. Public Health Nutr.

[27] Park HJ, Park S, Kim JY (2022). Development of Korean NOVA food classification and estimation of ultra-processed food intake among adults: using 2018 Korea National Health and Nutrition Examination Survey. Korean J Community Nutr.

[28] Jung S, Park S, Kim JY (2022). Comparison of dietary share of ultra-processed foods assessed with a FFQ against a 24-h dietary recall in adults: results from KNHANES 2016. Public Health Nutr.

[29] Ingram DD, Malec DJ, Makuc DM, Kruszon-Moran D, Gindi RM, Albert M, Beresovsky V, Hamilton BE, Holmes J, Schiller J, Sengupta M (2018). National center for health statistics guidelines for analysis of trends. Vital Health Stat.

[30] Guyomard H, Darcy-Vrillon B, Esnouf C, Marin M, Russel M, Guillou M (2012). Eating patterns and food systems: critical knowledge requirements for policy design and implementation. Agric Food Secur.

[31] Hess JM, Jonnalagadda SS, Slavin JL (2016). What is a snack, why do we snack, and how can we choose better snacks? A review of the definitions of snacking, motivations to snack, contributions to dietary intake, and recommendations for improvement. Adv Nutr.

[32] Mattes RD (2018). Snacking: a cause for concern. Physiol Behav.

[33] Cho EB, Park HA, Kang J-H, Kim K, Cho YG, Choi D-H (2017). Snack consumption patterns and its nutritional significance in Korean elderly population: From the 2013–2014 Korea National Health and Nutrition Examination Survey. Korean J Health Promot.

[34] Monteiro CA, Cannon G, Lawrence M, Costa Louzada Md, Pereira Machado P (2019). Ultra-processed foods, diet quality, and health using the NOVA classification system.

[35] Korea Disease Control and Prevention Agency (2020). Korea Health Statistics 2019: Korea National Health and Nutrition Examination Survey (KNHANES VIII-1).

[36] Yang YS, Han BD, Han K, Jung JH, Son JW (2022). Obesity Fact Sheet in Korea, 2021: trends in obesity prevalence and obesity-related comorbidity incidence stratified by age from 2009 to 2019. J Obes Metab Syndr.

[37] Poobalan AS, Aucott LS, Clarke A, Smith WC (2014). Diet behaviour among young people in transition to adulthood (18–25 year olds): a mixed method study. Health Psychol Behav Med.

[38] Grech AL, Rangan A, Allman-Farinelli M (2017). Dietary energy density in the Australian adult population from national nutrition surveys 1995 to 2012. J Acad Nutr Diet.

[39] Ministry of Health and Welfare (2021). Dietary guidelines for Koreans.

[40] Popkin BM, Barquera S, Corvalan C, Hofman KJ, Monteiro C, Ng SW, Swart EC, Taillie LS (2021). Towards unified and impactful policies to reduce ultra-processed food consumption and promote healthier eating. Lancet Diabetes Endocrinol.

[41] Kim EK, Fenyi JO, Kim JH, Kim MH, Yean SE, Park KW, Oh K, Yoon S, Ishikawa-Takata K, Park J, Kim JH, Yoon JS (2022). Comparison of total energy intakes estimated by 24-hour diet recall with total energy expenditure measured by the doubly labeled water method in adults. Nutr Res Pract.

[42] Kye S, Kwon SO, Lee SY, Lee J, Kim BH, Suh HJ, Moon HK (2014). Under-reporting of energy intake from 24-hour dietary recalls in the Korean National Health and Nutrition Examination Survey. Osong Public Health Res Perspect.

[43] Gemming L, Ni Mhurchu C (2016). Dietary under-reporting: what foods and which meals are typically under-reported?. Eur J Clin Nutr.

[44] Willett W (2013). Nutritional epidemiology.

[45] Leech RM, Worsley A, Timperio A, McNaughton SA (2015). Characterizing eating patterns: a comparison of eating occasion definitions. Am J Clin Nutr.

[46] Gibney MJ, Forde CG, Mullally D, Gibney ER (2017). Ultra-processed foods in human health: a critical appraisal. Am J Clin Nutr.

[47] Moubarac JC, Parra DC, Cannon G, Monteiro CA (2014). Food classification systems based on food processing: significance and implications for policies and actions: a systematic literature review and assessment. Curr Obes Rep.

