# Supplementary material for: Eating patterns in Korean adults, 1998–2018: increased energy contribution of ultra-processed foods in main meals and snacks
Source: Eur J Nutr. 2023 Nov 24;63(1):279–89. doi: 10.1007/s00394-023-03258-x (PMC10799128; doi:10.1007/s00394-023-03258-x)
Supplement: Supplementary file 1 — Supplementary file1 (DOCX 57 KB) [file 394_2023_3258_MOESM1_ESM.docx]

**Online Supporting Information**

This appendix is a part of the original submission and has been peer reviewed.

Supplement to: Jung S, Kim JY, and Park S. Eating patterns in Korean adults, 1998-2018: Increased energy contribution of ultra-processed foods in main meals and snacks

**Supplemental Figure 1. Study participants flow chart, KNHANES, 1998, 2007-2009, and 2016-2018 (n=29,389)**

If ages not in 20-69 years

(*n* 31,575)

Self-reported heart diseases, cancers, liver cirrhosis, and/or renal failure at the time of examination

(*n* 1,459)

Pregnant or lactating females

(*n* 255)

KNHANES 1998, 2007-2009, 2016-2018

(*n 8*8,200)

Not pregnant or lactating females

(*n* 30,848)

Final analytic sample

(*n* 29,389)

Plausible energy intake

(500-5000 kcal/d)

(*n* 31,103)

20-69 years of age

(*n* 56,625)

Valid 24-hour dietary recall

(*n* 32,228)

Implausible energy intake (<500, >5000 kcal/d)

(*n* 1,125)

Incomplete 24-hour dietary recall

(*n* 24,397)

**Supplemental Table 1. Most frequently consumed food or ingredient items for each NOVA group across the survey cycle, stratified by eating occasion**

|  | 1998 | 2009 | 2018 |
| --- | --- | --- | --- |
| **Breakfast** |  |  |  |
| MPF | Garlic | Garlic | Garlic |
|  | Rice | Rice | Rice |
|  | Green onion | Green onion | Sesame |
|  | Pepper powder | Sesame | Onion |
|  | Radish | Pepper powder | Pepper powder |
| PCI | Salt | Salt | Sesame oil |
|  | Sesame oil | Sesame oil | Salt |
|  | Sugar | Sugar | Perilla oil |
|  | Soybean oil | Soybean oil | Soybean oil |
|  | Corn syrup | Corn syrup | Sugar |
| PF | Kimchi | Kimchi | Kimchi |
|  | Soybean paste (homemade) | Soy sauce (homemade) | Soy sauce (homemade) |
|  | Soy sauce (homemade) | Laver | Tofu |
|  | Tofu | Soybean paste (homemade) | Fried eggs |
|  | Red pepper paste | Tofu | Laver |
| UPF | Soy sauce (manufactured) | Soy sauce (manufactured) | Soy sauce (manufactured) |
|  | MSG | MSG | Soybean paste |
|  | Coffee creamer | Soybean paste | MSG |
|  | Noodle | Coffee (manufactured) | Red pepper paste (manufactured) |
|  | Fried fishcake | Red pepper paste (manufactured) | Plum syrup |
| **Lunch** |  |  |  |
| MPF | Garlic | Garlic | Garlic |
|  | Green onion | Green onion | Onion |
|  | Rice | Rice | Green onion |
|  | Pepper powder | Pepper powder | Sesame |
|  | Onion | Sesame | Pepper powder |
| PCI | Salt | Salt | Salt |
|  | Sesame oil | Sesame oil | Sesame oil |
|  | Sugar | Soybean oil | Soybean oil |
|  | Soybean oil | Sugar | Sugar |
|  | Corn syrup | Corn syrup | Corn syrup |
| PF | Kimchi | Kimchi | Kimchi |
|  | Red pepper paste | Soy sauce (homemade) | Soy sauce (homemade) |
|  | Soybean paste | Tofu | Tofu |
|  | Tofu | Laver | Kimchi, radish |
|  | Soy sauce (homemade) | Kimchi, radish | Pickled radish |
| UPF | Soy sauce (manufactured) | Soy sauce (manufactured) | Soy sauce (manufactured) |
|  | MSG | Red pepper paste (manufactured) | MSG |
|  | Fried fishcake | Soybean paste (manufactured) | Red pepper paste (manufactured) |
|  | Coffee creamer | MSG | Soybean paste (manufactured) |
|  | Ketchup | Coffee (manufactured) | Anchovy broth |
| **Dinner** |  |  |  |
| MPF | Garlic | Garlic | Garlic |
|  | Green onion | Green onion | Onion |
|  | Rice | Rice | Rice |
|  | Pepper powder | Pepper powder | Sesame |
|  | Onion | Onion | Pepper powder |
| PCI | Salt | Salt | Salt |
|  | Sesame oil | Sesame oil | Sesame oil |
|  | Sugar | Sugar | Soybean oil |
|  | Soybean oil | Soybean oil | Sugar |
|  | Corn syrup | Corn syrup | Perilla oil |
| PF | Kimchi | Kimchi | Kimchi |
|  | Soybean paste | Soy sauce (homemade) | Soy sauce (homemade) |
|  | Red pepper paste | Tofu | Tofu |
|  | Tofu | Soybean paste (homemade) | Pickled radish |
|  | Soy sauce (homemade) | Laver | Laver |
| UPF | Soy sauce (manufactured) | Soy sauce (manufactured) | Soy sauce (manufactured) |
|  | MSG | Red pepper paste (manufactured) | Red pepper paste (manufactured) |
|  | Fried fishcake | Soybean paste (manufactured) | MSG |
|  | Glass noodle | MSG | Soybean paste (manufactured) |
|  | Noodle | Ssamjang | Plum syrup |
| **Snack** |  |  |  |
| MPF | Tangerine | Apple | Apple |
|  | Milk | Tangerine | Milk |
|  | Persimmon | Garlic | Tangerine |
|  | Apple | Green onion | Garlic |
|  | Green onion | Onion | Onion |
| PCI | Sugar | Sugar | Salt |
|  | Salt | Salt | Sugar |
|  | Soybean oil | Soybean oil | Soybean oil |
|  | Sesame oil | Sesame oil | Sesame oil |
|  | Corn syrup | Corn syrup | Corn syrup |
| PF | Coffee, instant | Coffee, instant | Coffee, instant |
|  | Kimchi | Kimchi | Kimchi |
|  | Citron tea | Tofu | Pickled radish |
|  | Red pepper paste | Soy sauce (homemade) | Tofu |
|  | Tofu | Pickled radish (homemade) | Soy sauce (homemade) |
| UPF | Coffee creamer | Coffee mix | Coffee mix |
|  | Soy sauce (manufactured) | Soy sauce (manufactured) | Soy sauce (manufactured) |
|  | Soju | Red pepper paste (manufactured) | Ketchup |
|  | Yogurt drink | Ketchup | Red pepper paste (manufactured) |
|  | Coke | Bread crumbs | Mayonnaise |

Abbreviation: MPF, unprocessed or minimally processed foods; PCI, processed culinary ingredients; PF, processed foods; UPF, ultra-processed foods; MSG, monosodium glutamate.
